# Characterization of the association between 8q24 and colon cancer: gene-environment exploration and meta-analysis

**DOI:** 10.1186/1471-2407-10-670

**Published:** 2010-12-04

**Authors:** Carolyn M Hutter, Martha L Slattery, David J Duggan, Jill Muehling, Karen Curtin, Li Hsu, Shirley AA Beresford, Aleksandar Rajkovic, Gloria E Sarto, James R Marshall, Nazik Hammad, Robert Wallace, Karen W Makar, Ross L Prentice, Bette J Caan, John D Potter, Ulrike Peters

**Affiliations:** 1 Division of Public Health Sciences, Fred Hutchinson Cancer Research Center, Seattle, USA; 2 Department of Internal Medicine, University of Utah Health Sciences Center, Salt Lake City, USA; 3 Genetic Basis of Human Disease Division, Translational Genomics Research Institute, Phoenix, USA; 4 Department of Obstetrics, Gynecology and Reproductive Sciences, University of Pittsburgh, USA; 5 Department of Obstetrics and Gynecology, University of Wisconsin, Madison, USA; 6 Department of Cancer Prevention and Population Sciences, Roswell Park Cancer Institute, Buffalo, USA; 7 Department of Medical Oncology, Cancer Center of Southeastern Ontario, Queen's University, Kingston, Canada; 8 Departments of Epidemiology and Internal Medicine, University of Iowa, College of Public Health, Iowa City, USA; 9 Division of Research, Kaiser Permanente Medical Care Program, Oakland, USA

## Abstract

**Background:**

Genome-wide association studies and subsequent replication studies have shown that single nucleotide polymorphisms (SNPs) in the chromosomal region 8q24 are associated with colorectal cancer susceptibility.

**Methods:**

We examined 11 SNP markers in the 8q24 region between 128.47 and 128.54 Mb, using a total of 1,987 colon cases and 2,339 controls who self-reported as white from two independent, well-characterized study populations. Analysis was performed separately within each study, and combined using random effects meta-analysis. Logistic regression was used to estimate odds ratios (ORs) and 95% confidence intervals (95% CIs) and to test for effect modification by known colon cancer risk factors. We also performed a meta-analysis combining our results with previous studies.

**Results:**

We observed evidence of association for four SNPs in low to high linkage disequilibrium (r^2 ^ranging from 0.18 to 0.93) localized in a 16.2 kb region defined by rs10505477 and rs1056368. The combined results for our two studies of colon cancer showed an OR of 1.10 (95% CI: 1.01-1.20, P_trend _= 0.023), and a meta-analysis of our results with previously reported studies of colon and colorectal cancer strongly support the association for this SNP (combined OR for rs6983267 = 1.21, 95% CI: 1.18-1.24, p = 5.5 × 10^-44^). We did not observe any notable evidence of effect modification by known colon cancer risk factors, and risk did not differ significantly by tumor site or stage.

**Conclusions:**

Our study confirms the association between polymorphisms on chromosome 8q24 and colon cancer risk and suggests that the susceptibility locus in region 8q24 is not strongly modified by various lifestyle, environmental, and demographic risk factors for colon cancer.

## Background

Colorectal cancer is the third leading cause of cancer and cancer death for both men and women in the United States [1]. At least 20%, and perhaps as much as one-third, of colorectal cancer is attributable to inherited factors [2]. High-penetrance mutations, such as those of the adenomatous polyposis coli gene (*APC*) and DNA mismatch-repair genes, account for < 5% of the cases and it is expected that a large proportion of the inherited susceptibility is likely to be explained by numerous low-penetrance variants in combination with susceptibility to diet, lifestyle, and environmental exposures. Numerous candidate gene studies have been performed for colorectal cancer, and a large number have detected associations. However, candidate gene studies have not been consistently replicated [3] and establishing the role of such low-penetrance variants with certainty has proved challenging [4].

The ability to conduct genome-wide association studies that include hundreds of thousands of single nucleotide polymorphisms (SNPs) has considerably improved this situation. Genome-wide scans and subsequent replication in other observational studies have shown that SNPs in the chromosomal region 8q24 are associated with prostate [5-14], breast [15], urinary bladder [16], and colorectal cancer [8,17-32]. For prostate cancer, three regions of 8q24 spanning in total a 430 Mb region were initially found to be independently associated, with recent studies identifying two additional regions [14,33]. In contrast, for colorectal cancer, statistically significant associations were initially reported only for an approximately 60 kb region of high linkage disequilibrium between chromosomal location 128.475 and 128.545 Mb. Although the strength of association between genetic variation in this region and colorectal cancer is modest, with reported per-allele odds ratios (ORs) ranging from 1.11 to 1.28, the region may contribute importantly to the susceptibility of colorectal cancer due to the high frequency of the variant. The minor allele frequency of the variant showing highest significance (rs6983267) is about 49-50% in European and European American populations [21,29,32]; 85-90% in African American populations [9,22]; and 30-35% in Japanese, Japanese American and Native Hawaiian populations [9,24].

In this paper, we followed up these intriguing findings by examining 11 SNP markers in the 8q24 region between positions 128.47 and 128.54 Mb in two independent, well-characterized study populations. In addition, we performed a meta-analysis of the rs6983267 variant in relation to risk of colon and colorectal cancer. Further, we characterized the epidemiologic architecture of this susceptibility locus by testing for interactions with known environmental risk factors for colorectal cancer, and by examining associations by tumor stage and site.

## Methods

The study uses colon cancer cases and controls from two previously described study populations: a multi-center population-based case-control study focusing on Diet, Activity and Lifestyle (DALS) and colon cancer [34] and a nested case-control study of colon cancer within the Women's Health Initiative (WHI) Observational Study [35-38].

Cases and controls in the multi-center DALS were recruited from three sites: the Kaiser Permanente Medical Care Program (KPMCP) of Northern California; an eight-county area in Utah; and the metropolitan Twin Cities area of Minnesota [34]. Cases were eligible if they were between 30-79 years of age at the time of diagnosis, with first primary colon cancer (International Classification of Diseases for Oncology [ICD-O] Ed.2. codes 18.0 and 18.2-18.9) between October 1991 and September 1994, and displayed sufficient mental competence to complete the interview. Cases with tumors in the rectosigmoid junction or rectum and cases with a pathology report indicating Familial Adenomatous Polyposis, Crohn's disease, or ulcerative colitis were excluded. A rapid-reporting system was used to identify all incident cases, with a majority being interviewed within 4 months of diagnosis. Response and participation proportions have been described previously [34]. Of the cases who were asked to participate in the study, 76% (n = 1,993) cooperated, of whom 83% (n = 1,676) provided a blood sample for DNA extraction.

Controls in DALS were frequency matched to cases by 5-year age groups and sex. Controls from KPMCP were randomly selected from membership lists. In Utah, controls who were under 65 years of age were randomly selected from lists generated using random-digit dialing and driver license lists, and those 65 years of age and older were randomly selected from Health Care Financing Administration lists. In Minnesota, control participants were identified from drivers license or state identification lists. Of the controls asked to participate, 64% (n = 2,410) cooperated, of whom 85% (n = 2,004) provided a blood sample for DNA extraction. Our primary analysis is restricted to 1,461 cases and 1,813 controls who self-reported as white.

The WHI Observational Study (OS), a prospective cohort study conducted in conjunction with the WHI randomized Clinical Trials (CT), recruited 93,676 postmenopausal women living in proximity to 40 clinical centers located across the USA [35-38]. Between Oct. 1, 1993, and Dec. 31, 1998, women aged 50 to 79 years were recruited into the WHI, mostly by age- and women-targeted mass mailings and announcements in the media. Exclusion criteria for both the OS and CT included participation in existing randomized trials, medical conditions predictive of a survival of less than 3 years, and conditions inconsistent with study participation and adherence. The clinical trials had additional eligibility requirements and the OS is substantially comprised of women who were ineligible for the CT or who did not want to be randomly assigned to an intervention. Cancer outcomes were initially assessed annually by mail or phone questionnaires. Self-reported colon cancer was adjudicated by trained physicians based on medical records and pathology reports at the clinical centers and centrally confirmed by blinded review at the WHI Coordinating Center [35]. Within this cohort, we conducted a nested case-control study among women who provided a blood sample for DNA extraction and did not refuse genetic testing. The censor date was September 12 2005. At that time, the mean follow-up time was 7.6 years with 2.2% lost to follow-up. Cases were defined as invasive incident colon cancer cases (ICD-O site codes 153.0-153.4 and 153.6-153.9). Control participants were randomly selected and individually matched on age at screening, enrollment date, ethnicity (white, African American, Hispanic, other), hysterectomy status, and prevalent condition at baseline using risk set sampling. Our primary analysis is restricted to 526 matched pairs of colon cancer cases and controls, representing all cases diagnosed before September 12, 2005 who self reported as white and had genotype data available for both the case and their matched control.

All participants included in this study provided written informed consent, and the study was approved by the human subjects committee at each participating institution.

For the DALS study, in-person interviews were used to collect demographic, dietary, and lifestyle data [39,40]. Study participants were asked about their lifestyle during the year, two years prior to the date of diagnosis or selection. During the in-person interview, information was collected on dietary intake, physical activity, medical history and drug use, demographic factors, smoking, reproductive history (for women), and family history of cancer and colorectal polyps. Dietary intake was ascertained using a diet-history questionnaire, which was modified from the questionnaire designed and validated for the Coronary Artery Risk Development in Young Adults (CARDIA) study [41].

For WHI, information on demographics, smoking and alcohol use, medical history, lifestyle/behavioral factors, family history of cancer, reproductive history, and details of physical activity, among other risk factors, were obtained by standardized self-administered questionnaires at baseline. Measurements for weight, height, waist, and hip were ascertained during a baseline physical examination. Details on medication usage, including non-steroidal anti-inflammatory drugs (NSAIDs), postmenopausal hormone (PMH), and vitamin and mineral supplements were collected from an interview-administered questionnaire during the baseline clinical visit. Dietary intake was assessed using a self-administered, 122-item Food Frequency Questionnaire at baseline.

Clinical and epidemiologic data was harmonized across the two studies through a systematic evaluation of individual study questionnaires, protocols, and data dictionaries. The reference date for DALS is two years prior to the date of diagnosis. The reference date for WHI is the baseline questionnaire date. We selected seven risk factors that are consistently shown to be associated with colon cancer risk. For family history, subjects were classified as having at least one 1^st ^degree relative (parents, siblings and children) with colorectal cancer versus no 1^st ^degree relatives with colorectal cancer. Non-smokers were defined as individuals smoking less than 100 cigarettes in their lifetime, and smokers were classified as either current or former smokers. Body mass index (BMI; kg/m^2^) was calculated based on baseline measurements of height and weight for the WHI study, and on self-report of height and weight on a reference date (~2 years before diagnosis for cases and interview for controls) in DALS. Our examination of effect modification by BMI was restricted to men and estrogen-positive women (e.g. premenopausal women and postmenopausal women currently taking PMH). For physical activity, subjects were divided into those with one or more hour of vigorous/strenuous leisure physical activity per week, and those without at least one hour of vigorous/strenuous leisure physical activity per week. For NSAID use, we divided subjects into current NSAID users versus not-current NSAID users. Effect modification by PMH was examined only in post-menopausal women and we compared ever versus never use of PMH. Current alcohol consumption was dichotomized to less than one drink per week versus one or more drinks per week.

Genotyping was performed using genomic DNA extracted from peripheral blood lymphocytes or from immortalized cell lines. The genotyping lab was blinded to case/control status and duplicate quality-control samples were interspersed among plates (341 duplicates from 226 different subjects). All genotyping was performed by MALDI-TOF mass spectrometry on the Sequenom MassARRAY Compact platform using the iPLEX Gold (low-plex) reaction. In total, we received clear genotyping results for 11 out of 14 SNPs. The remaining three SNPs (rs7002225, rs6998061 and rs6999921) were excluded for having discordances with the HapMap positive-control samples and/or severe departures from HWE (p < 1 × 10^-4^). In all three cases, one of the homozygous genotype calls was missing. The call rate was > 95% for 10 out of the 11 SNPs, and the 11^th ^SNP (rs6983267) had a call rate of 94.8%. Blinded duplicates displayed > 99% concordance for all SNPs. Using an exact test, the allele frequencies among controls for self-reported whites did not deviate from HWE for any SNP at p = 0.01. HWE for all SNPs for both the self-reported whites and the full population are presented in Additional file 1.

To estimate the association between genetic variants in the 8q24 region and colon cancer risk we calculated odds ratios (ORs) and 95% confidence intervals (95% CIs) using a two-stage pooled analysis, which allow us to combine results for the two studies, even though they have different designs [42,43]. Stage 1 used a model appropriate to each study design with study-specific confounders. Stage 2 used a random effects meta-analysis to combine the results for the two studies. For the individually matched data in the WHI nested case-control study, we used conditional logistic regression analysis to account for matching. For DALS data we used logistic regression adjusted for age, sex, and study center. Because of potential issues of population stratification when including non-white subjects in each study, we restricted our primary analyses to subjects who self-reported as white. Individual SNPs were coded based on the log-additive model, including a single variable, coded as the number of copies of the minor allele. Stage 2 combined the individual study-specific adjusted log-ORs using a linear mixed-effects model, which were then converted to ORs and 95% CIs. We also examined each SNP using a dominant and recessive model. Haplotype frequencies for cases and controls were compared using the HAPSTAT software [44]. Although we tested 11 SNPs, we did not adjust for multiple testing as this study is set up as a replication and fine-mapping study to confirm previous findings and narrow down the specific region of association. However it is important to evaluate our findings in light of the number of SNPs tested. A conservative Bonferroni correction for 11 SNPs would lead to a p-value threshold of 0.05/11 = 0.0045.

For SNPs that showed associations at alpha level 0.05 and no evidence of between-study heterogeneity, we explored interactions between genetic variations and known risk factors for colon cancer. Specifically, we conducted stratified analyses and tests for multiplicative interaction with inclusion of cross-product terms in the regression models. The statistical significance of interaction terms was assessed using a likelihood-ratio test comparing the full model including the interaction term to the nested model that included only the main effects and adjustment variables. We also did a combined case-only and case-control analysis to examine evidence for gene-environment interactions [45].

Because risk factors for colon cancer can differ by anatomic site, we used polytomous regression analysis to examine the association between variants and cancer subtypes defined by site (proximal colon: cecum, ascending colon, hepatic flexure of colon and transverse colon; distal colon: splenic flexure, descending colon, and sigmoid) and by SEER staging categories (localized, regional, or distant). In addition to polytomous regression, we also used the generalized odds ratio to examine stage, ranking localized, regional and distant disease in that order [46].

We conducted a meta-analysis including all published studies of the 8q24 variant rs6983267 and colorectal cancer. Because rs6983267 and rs10505477 are in high linkage disequilibrium (LD; r^2 ^= 0.93 in the HapMap CEU population), and because some studies have used them as tags for one another [32], we also searched for studies that examined the rs10505477 variant. Since it may be argued that high LD in HapMap does not justify switchability of the two alleles, we present the results for rs6983267 only as our primary analysis. Studies were identified through searches of PubMed and Web of Science using the search term "8q24" and either "colon cancer" or "colorectal cancer". We also examined Web of Science for all papers citing the original paper by Haiman and colleagues [8]. The last search was conducted on March 31, 2010. All papers were screened by one investigator (CMH), to assess whether they had association level data in a sample of at least 100 cases and 100 unrelated controls. Family based studies, including sib-pair analysis were excluded. No other additional screening or eligibility criteria were used. For each paper we extracted the following information into a table: author name, year of publication, geographic location, race/ethnicity, sample size and reported allelic or log-additive OR and 95% CI. Risk estimates from individual studies were combined and the corresponding summary 95% CI and p-values were obtained under a random-effects meta-analysis model [47,48]. Forest plots were used to display the results from individual studies, as well as the summary results. The statistical significance of between-study heterogeneity was evaluated using Cochran's Q statistic [47-49]. If the p-value was less than 0.10, the heterogeneity was considered statistically significant. We also quantified heterogeneity using the I^2 ^metric. I^2 ^takes values between 0 percent and 100 percent, with higher values indicating higher levels of heterogeneity [50]. Potential bias was assessed by comparing results for small and large studies using Egger's test and visual inspection of funnel plots [51], and we examined the trend in risk estimates over time using cumulative meta-analysis [52]. As a sensitivity analysis, we pre-specified that we would perform a meta-analysis combining studies that looked at rs6983267 and rs10505477, in addition to our primary meta-analysis only of studies examining the rs6983267 SNP. Because of the differences in allele frequencies observed across populations [9], we present meta-analysis results both for all studies combined, and for studies stratified by primary race/ethnicity. We also performed an *ad hoc *analysis including the study that used a sib-pair analysis and did not meet our initial inclusion criteria [26].

## Results

Table 1 shows characteristics of the study participants. By design, the WHI study comprises only female subjects and is restricted to subjects over age of 50. The DALS study had 53.1% male controls and 56.3% male cases, with 91.5% of controls and 92.7% of cases over the age of 50. The two studies show similar patterns for smoking and BMI, with both studies showing a higher proportion of cases who ever smoked and who had BMI > 25. DALS shows a greater difference between cases and controls on family history of colorectal cancer and recent NSAID use, with a higher frequency of a positive family history in cases and a higher frequency of NSAID use in the controls. Both studies show a slightly higher proportion of cases with < 1 hour of physical activity per week and a higher proportion of female controls using PMH, with higher overall physical activity levels in DALS and higher PMH use in the WHI study. For DALS, the regional distribution of cases was 44.8% from Northern California, 38.6% from Minnesota and 16.5% from Utah, and the distribution of controls was 37.5% from Northern California, 43.6% from Minnesota and 18.9% from Utah. For WHI, the regional distribution of cases was 23.7% from the Midwest, 27.2% from the North East, 21.5% from the South and 27.6% from the West, and the distribution of controls was 25.4% from the Midwest, 23.8% from the North East, 22.5% from the South and 28.3% from the West.

**Table 1:** Characteristics of study participants, by study

	DALS		WHI	
	
	Controls	Cases	Controls	Cases
Characteristic	n (%)	n (%)	n (%)	n (%)
**N**	1813	1461	526	526
**Gender**				
Male	963 (53.1)	822 (56.3)	0	0
Female	850 (46.9)	639 (43.7)	526 (100)	526 (100)
**Age**				
30-39	40 (2.1)	21 (1.4)	0	0
40-49	116 (6.4)	86 (5.9)	0	0
50-59	292 (16.1)	270 (18.5)	72 (13.7)	73 (13.9)
60-69	622 (34.3)	507 (34.7)	229 (43.5)	229 (43.5)
70-79	743 (41.0)	577 (39.5)	225 (42.8)	224 (43.6)
**Family History of CRC**				
No 1st degree relative	1637 (90.3)	1213 (83.0)	384 (80.5)	381 (77.1)
≥1 1st degree relatives	176 (9.7)	248 (17.0)	93 (19.5)	113 (22.9)
**Cigarette Smoker**				
Never	852 (47.0)	597 (41.0)	252 (48.2)	233 (44.8)
Former	715 (39.4)	649 (44.5)	240 (45.9)	252 (48.5)
Current	246 (13.6)	212 (14.5)	31 (5.9)	35 (6.8)
**BMI**				
< 25	720 (39.8)	485 (33.3)	199 (38.3)	186 (35.8)
≥25	1090 (60.2)	971 (66.7)	320 (61.7)	334 (64.2)
**Physical Activity**				
< 1 hour per week	1089 (60.1)	937 (64.2)	251 (48.3)	255 (49.0)
≥1 hours per week	724 (39.9)	523 (35.8)	269 (51.7)	265 (51.0)
**NSAID use**				
Not Current User	1098 (60.0)	1018 (69.7)	362 (68.8)	367 (69.8)
Current User	715 (39.4)	443 (30.3)	164 (31.2)	159 (30.2)
**Hormone Replacement Therapy Use**				
Not Current User	503 (69.2)	422 (76.1)	311 (59.2)	345 (65.7)
Current User	224 (30.8)	133 (23.9)	214 (40.8)	180 (34.3)
**Alcohol consumption**				
< 1 drink a week	1025 (56.5)	825 (56.5)	298 (56.8)	324 (62.0)
≥1 drinks per week	788 (43.5)	636 (43.5)	227 (43.2)	199 (38.1)
**Tumor Stage**				
Localized	NA	538 (38.5)	NA	212 (40.3)
Regional	NA	702 (50.2)	NA	228 (43.4)
Distant	NA	117 (8.4)	NA	67 (12.7)
Missing/Unknown	NA	103 (2.9)	NA	19 (3.6)
**Tumor Site**				
Distal Colon	NA	720 (49.3)	NA	143 (27.2)
Proximal Colon	NA	705 (48.3)	NA	361 (68.6)
Missing	NA	36 (2.5)	NA	22 (4.2)

DALS = Diet Activity and Lifestyle Study; WHI = Women's Health Initiative; N = total sample size; CRC = colorectal cancer; BMI = Body mass index; NSAID = Non-steroidal anti-inflammatory drugs

We genotyped 11 SNPs, all of which were in HWE within the control population of subjects who self-reported as white for both studies (p-value for exact test > 0.05). Four SNPs (rs10505477, rs10808555, rs6983267 and rs10956368) showed an increased risk of colon cancer at the alpha = 0.05 level in the combined analysis of individuals who self-report as white, with ORs ranging from 1.1 to 1.3 (Table 2). None of the SNPs would be significant if we used a correction for multiple testing for the 11 SNPs. Two of the four SNPs (rs10505477 and rs6983267) were also associated in DALS alone at the alpha = 0.05 level, and none were statistically significantly associated in the analysis restricted to the WHI samples (Table 1). All four SNPs had a similar magnitude of association across the two studies, with an I^2 ^measure of 0 and heterogeneity p-values > 0.5, indicating no evidence of between-study heterogeneity. A fifth SNP (rs9297756) was associated with an increased risk in DALS (OR = 1.22; 95% CI: 1.07-1.39) but showed a trend toward decreased risk in the WHI (OR = 0.93; 95% CI: 0.74-1.18). This SNP showed high between-study heterogeneity (I^2 ^= 78.4%, heterogeneity p = 0.032). We did not observe any additional significant SNPs when we considered dominant or recessive models of inheritance. Further, the haplotype frequencies did not show statistically significant differences between cases and controls (see Additional file 2).

**Table 2:** Associations between 8q24 SNPs and risk of colon cancer

			DALS						WHI						Combined		
					
SNP	Location	Allele*	MAF	Genotype	Cases	Controls	OR (95% CI)	p	MAF	Genotype	Cases	Controls	OR (95% CI)	p	OR (95% CI)	p	I^2^
rs16902148	128476181	G→A	0.05	GG	1328	1615	1 (Ref)		0.05	GG	595	589	1 (Ref)				
				GA	128	184	0.86 (0.68-1.09)			GA	46	57	0.81 (0.54-1.21)				
				AA	2	3	0.84 (0.14-5.05)			AA	1	3	0.33 (0.03-3.18)				
				log additive			0.86 (0.68-1.08)	0.203		log additive			0.75 (0.50-1.13)	0.168	0.83 (0.68-1.02)	0.073	0.0%
																	
rs10505477	128476625	C→T	0.49	CC	307	424	1 (Ref)		0.50	CC	144	162	1 (Ref)				
				CT	741	912	1.13 (0.95-1.35)			CT	323	328	1.10 (0.84-1.44)				
				TT	405	461	**1.23 (1.01-1.50)**			TT	169	156	1.21 (0.88-1.65)				
				log additive			**1.11 (1.00-1.22)**	**0.045**		log additive			1.08 (0.91-1.27)	0.384	**1.10 (1.01-1.20)**	**0.026**	0.0%
																	
rs10808555	128478693	G→A	0.33	GG	604	797	1 (Ref)		0.33	GG	278	285	1 (Ref)				
				GA	671	804	1.10 (0.95-1.28)			GA	279	293	0.97 (0.77-1.23)				
				AA	182	198	1.21 (0.97-1.52)			AA	81	67	1.24 (0.86-1.79)				
				log additive			1.10 (0.99-1.22)	0.067		log additive			1.08 (0.91-1.28)	0.395	**1.10 (1.00-1.20)**	**0.047**	0.0%
																	
rs6983267	128482487	T→G	0.48	TT	284	400	1 (Ref)		0.49	TT	137	149	1 (Ref)				
				TG	740	899	1.17 (0.97-1.40)			TG	303	326	1.00 (0.76-1.33)				
				GG	424	486	**1.23 (1.00-1.51)**			GG	174	158	1.19 (0.87-1.63)				
				log additive			**1.11 (1.00-1.22)**	**0.048**		log additive			1.09 (0.92-1.29)	0.336	**1.10 (1.01-1.20)**	**0.023**	0.0%
																	
rs10956368	128492832	C→T	0.41	CC	472	634	1 (Ref)		0.39	CC	218	236	1 (Ref)				
				CT	718	859	1.13 (0.96-1.32)			CT	295	316	1.00 (0.78-1.28)				
				TT	260	301	1.16 (0.95-1.43)			TT	115	91	1.37 (0.98-1.91)				
				log additive			1.09 (0.98-1.20)	0.100		log additive			1.12 (0.94-1.32)	0.201	**1.10 (1.01-1.20)**	**0.035**	0.0%
																	
rs7005829	128504126	C→T	0.29	CC	703	925	1 (Ref)		0.28	CC	350	328	1 (Ref)				
				CT	624	722	1.14 (0.99-1.32)			CT	237	270	0.82 (0.65-1.03)				
				TT	129	152	1.12 (0.86-1.44)			TT	51	48	1.00 (0.66-1.53)				
				log additive			1.09 (0.98-1.21)	0.113		log additive			0.94 (0.78-1.13)	0.523	1.03 (0.90-1.19)	0.647	**53.4%**
																	
rs9297756	128509349	C→T	0.15	CC	980	1290	1 (Ref)		0.16	CC	462	450	1 (Ref)				
				CT	428	458	**1.23 (1.05-1.44)**			CT	158	181	0.84 (0.65-1.08)				
				TT	46	42	1.42 (0.92-2.17)			TT	15	11	1.36 (0.62-3.01)				
				log additive			**1.22 (1.07-1.39)**	**0.004**		log additive			0.93 (0.74-1.18)	0.570	1.08 (0.83-1.41)	0.547	**78.4%**
																	
rs12334695	128523110	T→C	0.38	TT	522	684	1 (Ref)		0.38	TT	252	245	1 (Ref)				
				TC	678	833	1.08 (0.93-1.26)			TC	286	304	0.90 (0.71-1.15)				
				CC	235	268	1.16 (0.94-1.43)			CC	95	89	1.04 (0.74-1.46)				
				log additive			1.08 (0.97-1.19)	0.148		log additive			1.01 (0.85-1.19)	0.937	1.06 (0.97-1.16)	0.186	0.0%
																	
rs10109622	128527333	C→T	0.23	CC	896	1077	1 (Ref)		0.23	CC	393	388	1 (Ref)				
				CT	478	622	0.92 (0.79-1.07)			CT	203	220	0.91 (0.72-1.15)				
				TT	83	99	1.02 (0.76-1.39)			TT	39	38	1.01 (0.63-1.62)				
				log additive			0.96 (0.86-1.08)	0.537		log additive			0.99 (0.82-1.20)	0.954	0.97 (0.88-1.07)	0.512	0.0%
																	
rs10094059	128530789	G→C	0.24	GG	847	1030	1 (Ref)		0.25	GG	346	378	1 (Ref)				
				GC	523	677	0.94 (0.81-1.08)			GC	258	221	**1.28 (1.01-1.61)**				
				CC	87	93	1.13 (0.83-1.53)			CC	41	55	0.82 (0.53-1.26)				
				log additive			0.99 (0.88-1.11)	0.908		log additive			1.01 (0.84-1.22)	0.888	1.00 (0.90-1.10)	0.929	0.0%
																	
rs7841264	128535996	C→T	0.18	CC	1009	1190	1 (Ref)		0.17	CC	449	444	1 (Ref)				
				CT	395	546	0.85 (0.73-0.99)			CT	166	174	0.95 (0.74-1.22)				
				TT	50	59	1.01 (0.68-1.48)			TT	15	22	0.68 (0.34-1.33)				
				log additive			0.90 (0.79-1.03)	0.118	0.17	log additive			0.93 (0.75-1.16)	0.526	0.91 (0.81-1.02)	0.095	0.0%

* Reference allele is listed first.  Reference allele is the common allele for all SNPs but rs10505477 and rs6983267.  Those two SNPs have minor allele as reference for better comparison with previously published studies. DALS=Diet, activity and lifestyle study; WHI= Women's Health Intitiative; MAF=minor allele frequency; p=p-value from test of trend with genotype coded 0/1/2. I-squared measures between study heterogeneity. Associations significant at alpha level=0.05 are shown in bold.

**Figure 1:**
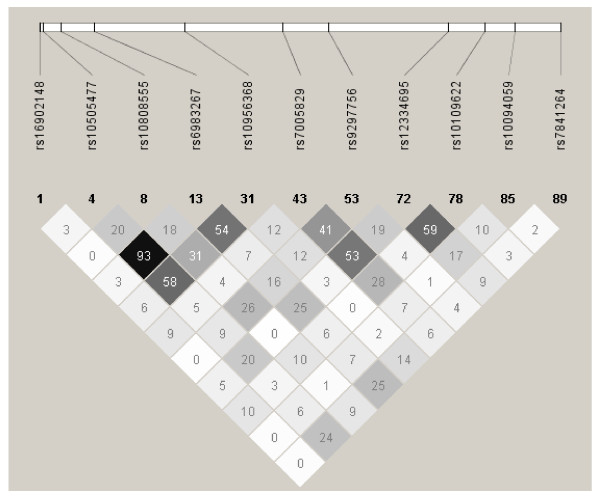
**Linkage disequilibrium in the 8q24 region between 128.47 and 128.54 Mb**. Linkage disequilibrium coefficients (r^2^) between SNPs in the 8q24 region between 128.47 and 128.54 Mb. As previously reported, r^2 ^is high for rs6983267 and rs10505477.

The four SNPs with a statistically significant association and low between-study heterogeneity are located in a 16,207 bp region (between positions 128,476,625 and 128,492,832) and show a range from low to high linkage disequilibrium (r^2 ^range: 0.18-0.93). LD was strongest between rs6983267 and rs10505477 (r^2 ^= 0.93) (Figure 1). These SNPs show slightly stronger evidence for association in men compared to women (see Additional file 3). For rs6983267, the OR for men is 1.16 (95% CI: 1.02-1.33, p = 0.03) and the OR for women (WHI and DALS combined) is 1.07 (95% CI: 0.97-1.87, p = 0.20). However, this is not a statistically significant difference in either DALS alone, or in the combined sample. Results were also similar when we included subjects that did not self-report as white (126 cases and 126 controls for WHI and 139 cases and 135 controls for DALS), with an OR of 1.12 (95% CI: 0.97-1.30, p = 0.14) for WHI and 1.12 (95% CI: 1.02-1.23, p = 0.02) for DALS.

Some of the SNPs showed differences in strength of association when stratified by colon cancer risk factors. For example, if we look at effect modification by BMI for rs10808555, there is a trend towards stronger association between the SNP in males and estrogen-positive women with a high BMI (OR = 1.19; 95% CI: 1.03-1.18) than in males and estrogen-positive women with a low BMI (OR = 0.96; 95% CI: 0.79-1.16, p-interaction = 0.08); however, we did not observe any evidence for statistically significant gene-environment interactions (see Additional file 3). We had similar results in the case-only analysis (results not shown). There was a slight tendency for the associations to be stronger in distant-stage, rather than localized or regional, cancers but, again, these differences were not larger than would be expected by chance (see Additional file 4). Our stage analysis was also not significant when we used the generalized OR. The associations did not differ notably by tumor site. One SNP, rs10109622 showed a potential association between the minor allele and decreased risk of proximal colon cancer (OR = 0.88; 95% CI: 0.77-0.99), with a non-significant odds ratio greater than 1.0 for distal colon cancer (OR = 1.07; 95% CI: 0.75-1.22), but this finding is not significant in either subsite once you adjust for multiple testing (see Additional file 5).

**Figure 2:**
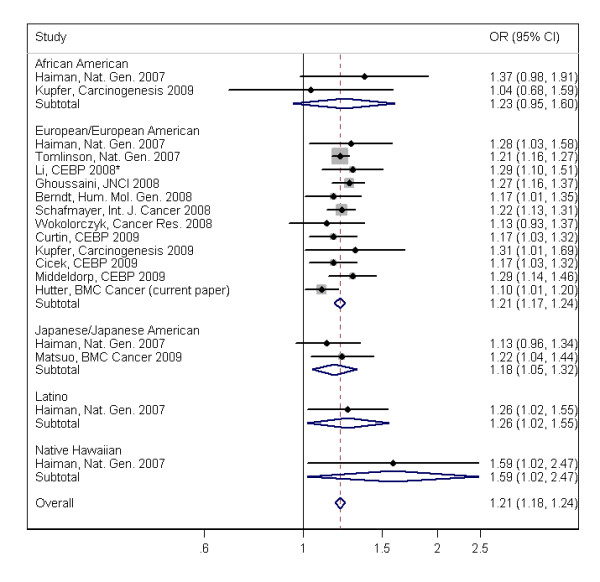
**Meta-analysis of the association between colorectal cancer and the 8q24 SNP rs6983267**. Forest plot of effect sizes and inverse-variance pooled estimates of the association between colorectal cancer risk and 8q24 SNP rs6983267. The symbol size indicates the weight for each study, and lines indicate the confidence intervals. Studies are grouped by race/ethnicity. One study (indicated by an asterisk) reported more than one racial/ethnic group, but is grouped with the European/European American studies, since that is the primary group (the percentage Caucasian is 94-95% for both cases and controls). The study reported that similar results were found when they restricted to white/Caucasian subjects. Results for rs6983267 were consistent across studies (I^2 ^= 0%; p-value for test of heterogeneity = 0.75).

Results for the meta-analysis are shown in Figure 2. This analysis is based on our data along with that of 17 other studies from 13 publications identified through our literature review for rs6983267 [9,17-20,22-25,27,29-31]. One study was excluded because it used a sib-pair analysis [26]. When data were combined across all studies for rs6983267, there was strong evidence for an association between rs6983267 and colorectal cancer risk (OR = 1.21; 95% CI: 1.18-1.24; p = 5.5 × 10^-44^); with no evidence of between-study heterogeneity (I^2 ^= 0.0%; heterogeneity p = 0.746). There was also no evidence for heterogeneity between racial/ethnic groups (p = 0.76), although the number and size of studies was relatively small for non-European/European American populations. A similar association was found when we also included studies that examined the rs10505477 variant [21,32] (OR = 1.19; 95% CI: 1.17-1.22; p = 2.2 × 10^-53^; see Additional file 6); again, there was no evidence for between-study heterogeneity (I^2 ^= 0.0%; overall heterogeneity p = 0.540). Our results are also consistent with those reported for the rs7014346 SNP in this region (OR = 1.19; p = 8.6 × 10^-26^) [28]. There was no evidence of differences between large and small studies based on Eggar's test (p = 0.44) and visual inspection of funnel plots. The cumulative meta-analysis shows that while the original estimate is slightly higher, the estimates have held steady in the range of 1.21 to 1.23 (see Additional file 7). As a *post hoc *analysis, we also included the study by Poynter and colleagues [26]. This study had been excluded on pre-specified inclusion/exclusion criteria because it used a sib-pair analysis rather than unrelated cases and controls; however, this was also the only study we found that reported an estimated OR less than one (for the clinic based samples). Inclusion of both the population and clinic-based estimates from this study did not substantively change the estimate for association (OR = 1.19; 95% CI: 1.15-1.23; p = 5.1 × 10^-23^), but did increase overall heterogeneity (I^2 ^= 26.5%; heterogeneity p = 0.14; see Additional file 6).

## Discussion

This study confirms the previously reported association between the 8q24 SNP rs6983267 and risk of colon cancer. The strength of association observed in our combined examination of WHI and DALS is relatively modest (OR = 1.10; 95% CI: 1.01-1.20; p = 0.023); however it is consistent with other studies as shown in our meta-analysis for rs6983267 (OR: 1.21; 95% CI: 1.18-1.24; p = 5.5 × 10^-44^). We examined 10 additional SNPs in the region defined by 128.47 and 128.54 Mb and found the associated interval at alpha = 0.05 to be localized in a 16.2 kb region defined by rs10505477 and rs1056368. We did not observe any statistically significant interactions between the 8q24 genotypes and known colon and colorectal cancer risk factors, and risk did not differ by tumor site or tumor stage.

The results of our meta-analysis strongly support the association between the rs6983267 variant, and SNPs in LD with this variant, and colon and colorectal cancer. Similar results are seen in both discovery GWAS studies [28,29,32] and follow-up replication studies [17-27,30,31]. Although the allele frequency for rs6983267 varies across racial/ethnic groups, the magnitude of the association appears to be consistent for studies which include subjects that do not self-report as white [26,27], and studies of non-white populations (see Figure 2). However, to date, most studies have focused on European and European American populations and results for other populations are based on only a handful of relatively small studies. The current meta-analysis included studies of both colon and colorectal cancer, combined studies that selected on family history with studies that did not, and did not examine alternative modes of inheritance, other than an additive model. The studies that show the genotype contrasts are generally consistent with an additive model. There is no strong evidence for reporting bias for this meta-analysis based on both funnel plots and cumulative meta-analysis; however it is still possible that publication bias is preventing, or delaying, the publication of studies that are not replicating the association [52]. It is also possible that there is substantive bias, even in the presence of a symmetrical funnel plot [53]. As more studies are published, with more detailed information, it will be important to do additional meta-analyses that stratify on these factors, as well as on key clinical and environmental risk factors. It will also be important to continue to move forward with functional follow-up studies to fully elucidate the causal mechanism underlying this association.

Variants in the 8q24 region have been associated with numerous cancers in addition to colorectal cancer. The region was identified as a top hit in genome-wide association studies of prostate [5-10,12-14], breast [15], urinary bladder [16] and colorectal cancers [8,29,32]. Follow-up in studies of multiple cancers have confirmed these findings [20] and have found additional associations for kidney, thyroid, and larynx cancer [31], as well as cancers of the upper aerodigestive tract [54]. More refined characterization of the 8q24 region shows that there are multiple sub-regions, each defined by an underlying LD structure [20,55]. These sub-regions show remarkable allelic heterogeneity, with some regions influencing risk for more than one cancer and some cancers being influenced by variants from more than one region [9,20].

The region of 8q24 examined in this study was coined as "Region 3" in a description of the multiple independent regions impacting prostate cancer [9]. SNPs from Region 3 have consistently been replicated for colorectal cancer. Most studies examining SNPs outside of Region 3 do not find evidence for associations with colorectal cancer [17,20,56], although one study did observe a significant association in Region 5 [19] and another study saw an association in Region 1 [18]. Initially Region 3 was defined as a 60 kb region spanning 128.48-128.54 Mb [9]. However, our study and others have narrowed the region. Ghoussaini *et al. *[20] examined nine SNPs across 8q24, including four in Region 3 and narrowed the area showing association with colorectal cancer risk to the 20 kb region between 128.48-128.50. Schafmayer *et al. *[27] examined 45 SNPs across 8q24 with 19 SNPs falling in Region 3. Their findings appeared to narrow the colorectal cancer susceptibility region to a 17.3 kb region defined by rs10505477 (at position 128,476,625) and rs7014346 (at position 128,493,974). Notably, this narrower region overlaps with the 16.2 kb region that contains the four significantly associated SNPs in the findings we report here, defined by rs10505477 (at position 128,476,625) and rs1056368 (at position 128,492,832). In this paper we focused our meta-analysis on rs6983267 because it is the most frequently published SNP and the only variant with more than three publications. If studies of 8q24 SNPs other than rs6983267 continue to be published for colorectal cancer, it will be informative to perform meta-analyses of these other variants. Further fine mapping and functional studies of 8q24 and colorectal cancer should focus on variants in high LD with this narrow region.

We identified no statistically significant interactions between genetic variants in 8q24 and various known risk factors for colon cancer. Although some trends were observed, we acknowledge that we have examined a large number of factors, and our results should be interpreted in the context of the large number of tests performed. This finding of no significant interactions is consistent with our previous study of 2,587 colorectal adenoma and 547 colorectal cancer cases in European American populations, which also showed no statistically significant interactions between rs6983267 and smoking, family history, age, or sex [17], as well as a study of 481 colorectal cancer cases from Japan, which showed no statistically significant interactions between rs6983267 and smoking, drinking habits, folate consumption, BMI, family history and exercise [24]. Our finding of no difference based on family history of colorectal cancer is also consistent with a Polish case-control study of 779 colon cancer cases that showed no interaction between rs6983267 and family history [31], although that study did observe higher OR estimates for cases with a 1^st ^degree relative with cancer compared to those without at other cancer sites (prostate, breast, bladder, larynx and lung). In addition, a study of 438 cases from Utah, 405 from Sheffield and 249 from Leeds found notable differences in the strength of association between the US site and the two UK sites, suggesting potential gene-environment interactions; however, this observation was not explained by differences in family history or early onset of disease. Given the potential for differences between males and females, it is important to note that the study did not observe effect modification by sex [19]. These initial findings suggest that the susceptibility locus in region 8q24 is not strongly modified by plausible lifestyle, environmental, and demographic risk factors for colorectal cancer. However, the number of studies that have explored such interactions is small and may have been underpowered for subtle effect modification. For example, our study was powered to detect an interaction odds ratio of 1.3 or greater for an interaction between the 8q24 variants and a dichotomous trait with a population prevalence of 40 percent, which is similar to the frequency of the environmental factors included in this analysis (see Table 1). Given the low magnitude of association for this variant, it is also possible that there will be a low, but reproducible, interaction effect. Hence, the finding of no effect modification still requires further corroboration. In particular, larger follow-up studies should still consider potential effect modification by sex.

The 8q24 region is typically described as a gene desert [57]. The 8q24 locus contains two transcripts with open reading frames, which are both mapped to the region of LD at 8q24.21: DQ515897 is a gene with unknown function and DQ486513 is a putative pseudogene of the *POU5F1*, which encodes the transcription factor OCT4 [29]. However, the strongest candidate for 8q24 function has always been the v-myc myelocytomatosis viral oncogene homolog gene (*MYC*), located about 350 kb telomeric of rs10505477 and rs6983267 [58]. *MYC *is an important protooncogene, over-expressed in numerous tumors, including colorectal tumors. Two recent studies have provided several important insights into the functional role of the rs6983267 variant [59,60]. First, they showed preferential amplification of the haplotype containing the rs6983207 G risk allele in colorectal tumors. Second, they showed that the variant is located in a transcriptional enhancer and that the G allele has increased affinity for binding transcription factor 4 (TCF4). The TCF4 transcription factor plays a role in activating the transcription of Wnt target genes. Third, they showed that the rs6983267 region physically interacts with the *MYC *promoter region. Together, these results point to an impact on Wnt signaling and *MYC *expression as a strong candidate for the biological mechanisms connecting the rs6983267 variant to risk of colorectal cancer [57,59,60]. In considering this mechanism, it is important to note that, similar to an earlier study [32], the recent studies did not observe differential *MYC *expression in colon tumors based on 8q24 genotypes. However, the studies many not be sensitive to subtle effects that could be relevant for disease risk. Further, the studies do not capture gene expression throughout cancer development [57].

A major strength of this analysis was that we examined the genetic variation in the chromosome 8q24 region between 128.47 and 128.54 Mb in two well-characterized studies with rich environmental data. This allowed us to thoroughly investigate potential gene-environment interactions and to examine the association by tumor site and location. The two studies differ on several key features. Most notably, the WHI is a matched nested case-control study of older women, whereas DALS is a population-based case-control study of both women and men. However, with the exception of rs9297756, the results were very consistent across the two studies. Further, the rs9297756 finding does not appear to be replicated in other studies, indicating that it is likely to be a spurious finding in DALS rather than a true heterogeneous finding across the two populations. The studies also differed on how they assessed information on environmental risk factors. Notably, for WHI we used prospective ascertainment of baseline characteristics, where as the DALS study had retrospective ascertainment for a period two years before the diagnosis (cases) or selection (controls) date. There were also some differences in how variables were ascertained. For example, for NSAID use, DALS had detailed information on past use and duration from questionnaire data on all participants, while the WHI had information only for current NSAID users. We were able to compare data across studies by restricting our analysis to a comparison of current users at baseline or reference date and current non-users at baseline or reference date. It is possible that misclassification or lack of comparability is responsible for our null findings for gene-environment interactions. Future studies should also investigate potential gene-environment interactions in other well-characterized population-based studies.

## Conclusions

Our results from a multi-center case-control study and the WHI cohort replicate previous findings of an association between polymorphisms in a 16.2 kb region located between 128.4766 and 128.4928 Mb on chromosome 8q24 and colon cancer risk. Taken as a whole, the evidence for this association is consistent across studies and populations. Most studies performed to date have focused on European and European American populations. Additional replication in other ethnic and racial groups is merited; because of differences in LD patterns across populations, such studies should initially examine variation across the entire 8q24 region. Our results do not suggest evidence for gene-environment interactions underlying this association.

## Competing interests

The authors declare that they have no competing interests.

## Authors' contributions

CMH carried out the statistical analyses, coordinated the study and drafted the manuscript. MLS was a lead in the conception, design and coordination of the DALS study and provided substantive input into the analysis and manuscript writing. DJD oversaw the SNP selection and genotyping. JM performed the genotyping. KC coordinated the data management and delivery of the DALS data and assisted with manuscript preparation. LH participated in the design and analysis of the statistical methods. SAAB, AR, GES, JRM and NH participated in the design and coordination of the WHI study, and provided feedback on the design for this analysis and manuscript. RW participated in the design and coordination of the WHI study, and provided substantive input into the manuscript writing. KWM performed the DNA extraction, oversaw sample handing and management and assisted with SNP selection. RLP leads the WHI clinical coordinating center, and provided substantive input into the statistical methods and manuscript writing. BJC was a lead in the conception, design and coordination of the DALS study, participated in the design and coordination of the WHI study, and provided feedback on the design for this analysis and manuscript. JDP was a lead in the conception, design and coordination of the DALS study, helped conceive of the study and design for this analysis and manuscript, and also helped to draft the manuscript. UP conceived of the study and participated in its design and coordination, and helped to draft the manuscript. All authors read and approved the final version of this manuscript.

## Pre-publication history

The pre-publication history for this paper can be accessed here:

http://www.biomedcentral.com/1471-2407/10/670/prepub

## Supplementary Material

Additional file 1 **Supplemental table S1: Exact Hardy-Weinberg p-value in controls for self-reported whites and full population for each study**. Presents the HWE p-values for controls in WHI and DALS separately, showing both the full population (all) and subjects who self report as white. Click here for file

Additional file 2 **Supplemental table S2: Haplotype frequencies for cases and controls**. Presents the haplotype frequencies for cases, controls and full population for both DALS and WHI. Click here for file

Additional file 3 **Supplemental table S3a: Associations between rs10956368 and colorectal cancer stratified by CRC risk factors, by study population**. **Supplemental table S3b: Associations between rs6983267 and colorectal cancer stratified by CRC risk factors, by study population**. **Supplemental table S3c: Associations between rs10956368 and colorectal cancer stratified by CRC risk factors, by study population**. Each worksheet gives information on our investigation of gene-environment interactions using stratified analysis for a different SNP (rs10808555, rs6983267, rs10956368). Results are presented for WHI, DALS and the combined study. Click here for file

Additional file 4 **Supplemental table S4: Associations between 8q24 SNPs and colon cancer/colorectal cancer risk stratified by tumor stage**. Presents results by tumor stage (localized, regional and distant). Results are presented for WHI, DALS and the combined study. Click here for file

Additional file 5 **Supplemental table S5: Associations between 8q24 SNPs and colorectal cancer risk stratified by tumor site**. Presents results by tumor site (Total Colon, Distal Colon and Proximal Colon). Results are presented for WHI, DALS and the combined study. Click here for file

Additional file 6 **Supplemental figure S1: Meta-analysis of the association between colorectal cancer and the 8q24 SNPs rs6983267 and rs10505477 including sib-pair analysis**. Forest plot of effect sizes and inverse-variance pooled estimates of the association between colorectal cancer risk and 8q24 SNPs for a) both rs6983267 and rs10505477; and b) including the study by Poynter et al which used sib-pair analysis. Click here for file

Additional file 7 **Supplemental figure S2. Funnel plot and cumulative meta-analysis plots for meta-analysis**. Funnel plot of odds ratio (OR) vs. standard error of OR for studies included in the meta-analysis and cumulative meta-analysis plots for rs6983267 in European/European American populations. These diagnostic plots can be used to assess biases. Click here for file
