# Supplementary material for: Characterization of the association between 8q24 and colon cancer: gene-environment exploration and meta-analysis
Source: BMC Cancer. 2010 Dec 4;10:670. doi: 10.1186/1471-2407-10-670 (PMC3017062; doi:10.1186/1471-2407-10-670)
Supplement: Additional file 1 — Supplemental table S1: Exact Hardy-Weinberg p-value in controls for self-reported whites and full population for each study. Presents the HWE p-values for controls in WHI and DALS separately, showing both the full population (all) and subjects who self report as white. [file 1471-2407-10-670-S1.PDF]

Supplemental Table 1: Exact Hardy Weinberg p-value in controls for self-reported whites and full population for each study

	DALS		WHI	
SNP	White	All	White	All
rs16902148	0.4793	0.6268	0.1859	0.0283
rs10505477	0.5395	0.8911	0.7529	0.247
rs10808555	0.8736	0.7597	0.5351	1
rs6983267	0.7044	0.7477	0.4748	0.2716
rs10956368	0.7322	0.5412	0.4061	0.2682
rs7005829	0.5246	0.3992	0.4983	0.7196
rs9297756	0.854	0.7223	0.1803	0.2289
rs12334695	0.5821	0.5941	0.8005	0.4827
rs10109622	0.4614	0.1695	0.3728	0.023
rs10094059	0.1958	0.3927	0.072	0.0038
rs7841264	0.7544	0.7091	0.3283	0.1493
