# Supplementary material for: Characterization of the association between 8q24 and colon cancer: gene-environment exploration and meta-analysis
Source: BMC Cancer. 2010 Dec 4;10:670. doi: 10.1186/1471-2407-10-670 (PMC3017062; doi:10.1186/1471-2407-10-670)
Supplement: Additional file 2 — Supplemental table S2: Haplotype frequencies for cases and controls. Presents the haplotype frequencies for cases, controls and full population for both DALS and WHI. [file 1471-2407-10-670-S2.PDF]

Supplement Table 2: Haplotype frequencies for cases and controls.

	DALS			WHI		
haplotype	controls	cases	combined	controls	cases	combined
00000000000	0.0985	0.0819	0.0911	0.0966	0.0972	0.0972
00000000001	0.1374	0.1276	0.1327	0.1371	0.1142	0.1248
00000000010	0.1213	0.1257	0.1228	0.1448	0.133	0.1391
00000101100	0.0689	0.0698	0.069	0.0719	0.0608	0.0678
00100000010	0.024	0.0202	0.0226	0.0287	0.0273	0.0277
01010001100	0.0633	0.0624	0.0631	0.063	0.0667	0.0644
01010001101	0.0145	0.0093	0.0119	0.0115	0.0123	0.0119
01011000000	0.0446	0.0558	0.0497	0.0449	0.0634	0.055
01111000000	0.0792	0.0778	0.0785	0.0713	0.0704	0.071
01111000010	0.0685	0.0606	0.065	0.0531	0.0766	0.0658
01111111000	0.114	0.1339	0.1223	0.1222	0.1249	0.1217
11011101100	0.0399	0.0363	0.0389	0.0428	0.034	0.0374
