# Supplementary material for: Characterization of the association between 8q24 and colon cancer: gene-environment exploration and meta-analysis
Source: BMC Cancer. 2010 Dec 4;10:670. doi: 10.1186/1471-2407-10-670 (PMC3017062; doi:10.1186/1471-2407-10-670)
Supplement: Additional file 4 — Supplemental table S4: Associations between 8q24 SNPs and colon cancer/colorectal cancer risk stratified by tumor stage. Presents results by tumor stage (localized, regional and distant). Results are presented for WHI, DALS and the combined study. [file 1471-2407-10-670-S4.PDF]

Supplemental Table 4: Associations between 8q24 SNPs and colon cancer/colorectal cancer risk stratified by tumor stage

		DALS		WHI		Combined	
**SNP**		**OR (95% CI)**	**p**	**OR (95% CI)**	**p**	**OR (95% CI)**	**p**
rs16902148	Total	0.86 (0.68-1.08)	0.20	0.75 (0.50-1.13)	0.17	0.82 (0.67-1.00)	0.05
	Localized	0.91 (0.66-1.25)	0.55	0.78 (0.47-1.27)	0.32	0.86 (0.66-1.13)	0.29
	Regional	0.81 (0.60-1.09)	0.17	0.81 (0.49-1.34)	0.41	0.81 (0.63-1.05)	0.11
	Distant	0.63 (0.30-1.30)	0.21	0.79 (0.34-1.83)	0.58	0.69 (0.40-1.20)	0.19
rs10505477	Total	1.11 (1.00-1.22)	0.05	1.08 (0.91-1.27)	0.38	1.10 (1.01-1.20)	0.03
	Localized	1.05 (0.92-1.21)	0.47	1.17 (0.96-1.43)	0.12	1.09 (0.97-1.22)	0.13
	Regional	1.07 (0.94-1.21)	0.30	1.03 (0.84-1.27)	0.78	1.06 (0.95-1.18)	0.30
	Distant	1.38 (1.05-1.80)	0.02	0.99 (0.71-1.39)	0.95	1.20 (0.98-1.49)	0.08
rs10808555	Total	1.10 (0.99-1.22)	0.07	1.08 (0.91-1.28)	0.39	1.10 (1.00-1.20)	0.03
	Localized	1.05 (0.91-1.21)	0.50	1.11 (0.90-1.37)	0.33	1.07 (0.95-1.21)	0.25
	Regional	1.14 (1.00-1.29)	0.05	1.02 (0.82-1.26)	0.88	1.10 (0.99-1.23)	0.08
	Distant	1.24 (0.95-1.64)	0.12	0.96 (0.68-1.37)	0.84	1.13 (0.91-1.40)	0.28
rs6983267	Total	1.11 (1.00-1.22)	0.05	1.09 (0.92-1.29)	0.34	1.10 (1.01-1.20)	0.03
	Localized	1.06 (0.92-1.21)	0.45	1.18 (0.97-1.45)	0.10	1.10 (0.98-1.23)	0.11
	Regional	1.07 (0.94-1.21)	0.31	1.06 (0.86-1.30)	0.61	1.06 (0.96-1.18)	0.26
	Distant	1.40 (1.06-1.84)	0.02	0.95 (0.68-1.33)	0.77	1.20 (0.97-1.48)	0.10
rs10956368	Total	1.09 (0.98-1.20)	0.10	1.12 (0.94-1,32)	0.20	1.10 (1.01-1.20)	0.03
	Localized	1.05 (0.91-1.20)	0.51	1.19 (0.97-1.46)	0.09	1.09 (0.97-1.23)	0.13
	Regional	1.13 (1.00-1.28)	0.06	1.01 (0.82-1.25)	0.90	1.10 (0.99-1.22)	0.09
	Distant	1.20 (0.92-1.57)	0.18	1.21 (0.86-1.70)	0.27	1.20 (0.98-1.48)	0.08
rs7005829	Total	1.09 (0.98-1.21)	0.11	0.94 (0.78-1.13)	0.52	1.04 (0.95-1.14)	0.39
	Localized	1.06 (0.91-1.23)	0.44	0.89 (0.72-1.12)	0.33	1.01 (0.89-1.14)	0.90
	Regional	1.10 (0.96-1.25)	0.18	0.91 (0.72-1.15)	0.43	1.05 (0.93-1.17)	0.45
	Distant	1.19 (0.90-1.58)	0.22	1.13 (0.79-1.62)	0.51	1.18 (0.94-1.47)	0.15
rs9297756	Total	1.22 (1.07-1.39)	0.00	0.93 (0.74-1.18)	0.57	1.14 (1.02-1.28)	0.02
	Localized	1.23 (1.02-1.47)	0.03	0.97 (0.73-1.28)	0.81	1.15 (0.99-1.34)	0.08
	Regional	1.18 (1.00-1.39)	0.05	0.82 (0.61-1.10)	0.18	1.08 (0.93-1.24)	0.31
	Distant	1.55 (1.12-2.14)	0.01	1.13 (0.72-1.78)	0.59	1.40 (1.08-1.82)	0.01
rs12334695	Total	1.08 (0.97-1.19)	0.15	1.01 (0.85-1.19)	0.94	1.05 (0.96-1.14)	0.27
	Localized	1.04 (0.90-1.19)	0.62	0.98 (0.80-1.21)	0.86	1.02 (0.91-1.14)	0.75
	Regional	1.01 (0.89-1.15)	0.86	0.99 (0.80-1.22)	0.91	1.00 (0.90-1.12)	0.93
	Distant	1.30 (1.00-1.70)	0.05	1.11 (0.79-1.56)	0.54	1.23 (1.00-1.51)	0.06
rs10109622	Total	0.96 (0.86-1.08)	0.54	0.99 (0.82-1.20)	0.96	0.96 (0.87-1.06)	0.47
	Localized	0.93 (0.79-1.09)	0.36	0.91 (0.72-1.15)	0.42	0.92 (0.80-1.05)	0.21
	Regional	0.91 (0.79-1.06)	0.22	1.04 (0.82-1.31)	0.76	0.95 (0.83-1.07)	0.37
	Distant	0.95 (0.69-1.30)	0.75	0.94 (0.64-1.40)	0.78	0.95 (0.74-1.21)	0.65
rs10094059	Total	1.11 (0.95-1.30)	0.20	1.01 (0.84-1.22)	0.89	1.00 (0.91-1.11)	0.92
	Localized	1.11 (0.95-1.30)	0.20	1.11 (0.89-1.38)	0.36	1.11 (0.97-1.26)	0.12
	Regional	1.03 (0.89-1.19)	0.71	1.03 (0.82-1.29)	0.81	1.03 (0.91-1.16)	0.65
	Distant	0.70 (0.50-0.99)	0.05	1.18 (0.82-1.68)	0.38	0.89 (0.69-1.14)	0.35
rs7841264	Total	0.90 (0.79-1.03)	0.12	0.93 (0.75-1.16)	0.53	0.91 (0.81-1.02)	0.09
	Localized	0.87 (0.72-1.04)	0.13	0.82 (0.62-1.09)	0.17	0.86 (0.73-1.00)	0.04
	Regional	0.93 (0.79-1.09)	0.35	1.04 (0.80-1.37)	0.75	0.95 (0.83-1.09)	0.50
	Distant	1.05 (0.75-1.46)	0.79	0.65 (0.39-1.07)	0.09	0.89 (0.67-1.17)	0.40
