# Supplementary material for: Characterization of the association between 8q24 and colon cancer: gene-environment exploration and meta-analysis
Source: BMC Cancer. 2010 Dec 4;10:670. doi: 10.1186/1471-2407-10-670 (PMC3017062; doi:10.1186/1471-2407-10-670)
Supplement: Additional file 5 — Supplemental table S5: Associations between 8q24 SNPs and colorectal cancer risk stratified by tumor site. Presents results by tumor site (Total Colon, Distal Colon and Proximal Colon). Results are presented for WHI, DALS and the combined study. [file 1471-2407-10-670-S5.PDF]

Supplemental Table 5: Associations between 8q24 SNPs and colorectal cancer risk stratified by tumor site

SNP		DALS		WHI		Combined	
		OR (95% CI)	p	OR (95% CI)	p	OR (95% CI)	p
rs16902148	Colon	0.86 (0.68-1.08)	0.20	0.75 (0.50-1.13)	0.17	0.82 (0.67-1.00)	0.05
	Distal	0.97 (0.73-1.28)	0.80	0.83 (0.45-1.55)	0.57	0.94 (0.73-1.21)	0.64
	Proximal	0.74 (0.54-1.00)	0.05	0.71 (0.45-1.14)	0.16	0.73 (0.57-0.95)	0.02
rs10505477	Colon	1.11 (1.00-1.22)	0.05	1.08 (0.91-1.27)	0.38	1.10 (1.01-1.20)	0.03
	Distal	1.16 (1.02-1.31)	0.02	1.09 (0.85-1.41)	0.49	1.15 (1.02-1.28)	0.02
	Proximal	1.06 (0.94-1.20)	0.34	1.07 (0.89-1.29)	0.48	1.07 (0.96-1.18)	0.22
rs10808555	Colon	1.10 (0.99-1.22)	0.07	1.08 (0.91-1.28)	0.40	1.10 (1.01-1.21)	0.03
	Distal	1.07 (0.94-1.22)	0.29	1.00 (0.77-1.31)	0.99	1.06 (0.94-1.19)	0.34
	Proximal	1.16 (1.02-1.32)	0.03	1.11 (0.92-1.35)	0.28	1.15 (1.03-1.28)	0.01
rs6983267	Colon	1.11 (1.00-1.22)	0.05	1.09 (0.92-1.29)	0.34	1.10 (1.01-1.20)	0.03
	Distal	1.14 (1.01-1.30)	0.04	1.11 (0.85-1.43)	0.44	1.14 (1.02-1.27)	0.03
	Proximal	1.08 (0.95-1.22)	0.24	1.08 (0.89-1.30)	0.44	1.08 (0.97-1.20)	0.15
rs10956368	Colon	1.09 (0.98-1.20)	0.10	1.12 (0.94-1.32)	0.20	1.10 (1.01-1.20)	0.03
	Distal	1.09 (0.96-1.23)	0.19	1.17 (0.90-1.51)	0.24	1.10 (0.98-1.23)	0.10
	Proximal	1.10 (0.97-1.25)	0.12	1.10 (0.91-1.32)	0.34	1.10 (0.99-1.22)	0.07
rs7005829	Colon	1.09 (0.98-1.21)	0.11	0.94 (0.78-1.13)	0.52	1.04 (0.95-1.14)	0.39
	Distal	1.12 (0.98-1.28)	0.10	0.94 (0.71-1.24)	0.65	1.08 (0.96-1.22)	0.21
	Proximal	1.06 (0.93-1.21)	0.40	0.94 (0.77-1.16)	0.58	1.02 (0.92-1.15)	0.67
rs9297756	Colon	1.22 (1.07-1.39)	0.00	0.93 (0.74-1.18)	0.57	1.14 (1.02-1.28)	0.02
	Distal	1.19 (1.01-1.41)	0.03	0.99 (0.69-1.40)	0.94	1.15 (0.99-1.33)	0.07
	Proximal	1.26 (1.07-1.48)	0.01	0.91 (0.70-1.19)	0.50	1.15 (1.00-1.32)	0.05
rs12334695	Colon	1.08 (0.97-1.19)	0.15	1.01 (0.85-1.19)	0.94	1.05 (0.96-1.14)	0.27
	Distal	1.14 (1.00-1.29)	0.04	1.05 (0.81-1.36)	0.70	1.12 (1.00-1.25)	0.05
	Proximal	1.00 (0.88-1.13)	0.99	0.99 (0.82-1.19)	0.90	1.00 (0.90-1.11)	0.94
rs10109622	Colon	0.96 (0.86-1.08)	0.54	0.99 (0.82-1.20)	0.95	0.96 (0.87-1.06)	0.47
	Distal	1.09 (0.94-1.25)	0.24	0.98 (0.73-1.32)	0.91	1.07 (0.95-1.22)	0.27
	Proximal	0.82 (0.70-0.95)	0.01	1.00 (0.81-1.24)	1.00	0.88 (0.77-0.99)	0.04
rs10094059	Colon	0.99 (0.88-1.11)	0.91	1.01 (0.84-1.22)	0.89	1.00 (0.91-1.11)	0.92
	Distal	0.97 (0.84-1.12)	0.70	0.80 (0.59-1.08)	0.14	0.94 (0.82-1.07)	0.34
	Proximal	1.04 (0.90-1.20)	0.61	1.11 (0.91-1.36)	0.32	1.06 (0.95-1.20)	0.30
rs7841264	Colon	0.90 (0.79-1.03)	0.12	0.93 (0.75-1.16)	0.53	0.91 (0.81-1.02)	0.10
	Distal	0.90 (0.77-1.06)	0.22	1.03 (0.74-1.44)	0.85	0.93 (0.80-1.07)	0.30
	Proximal	0.90 (0.77-1.06)	0.21	0.89 (0.69-1.14)	0.36	0.90 (0.78-1.03)	0.11
