# Supplementary material for: Characterization of the association between 8q24 and colon cancer: gene-environment exploration and meta-analysis
Source: BMC Cancer. 2010 Dec 4;10:670. doi: 10.1186/1471-2407-10-670 (PMC3017062; doi:10.1186/1471-2407-10-670)
Supplement: Additional file 6 — Supplemental figure S1: Meta-analysis of the association between colorectal cancer and the 8q24 SNPs rs6983267 and rs10505477 including sib-pair analysis. Forest plot of effect sizes and inverse-variance pooled estimates of the association between colorectal cancer risk and 8q24 SNPs for a) both rs6983267 and rs10505477; and b) including the study by Poynter et al which used sib-pair analysis. [file 1471-2407-10-670-S6.PDF]

A.

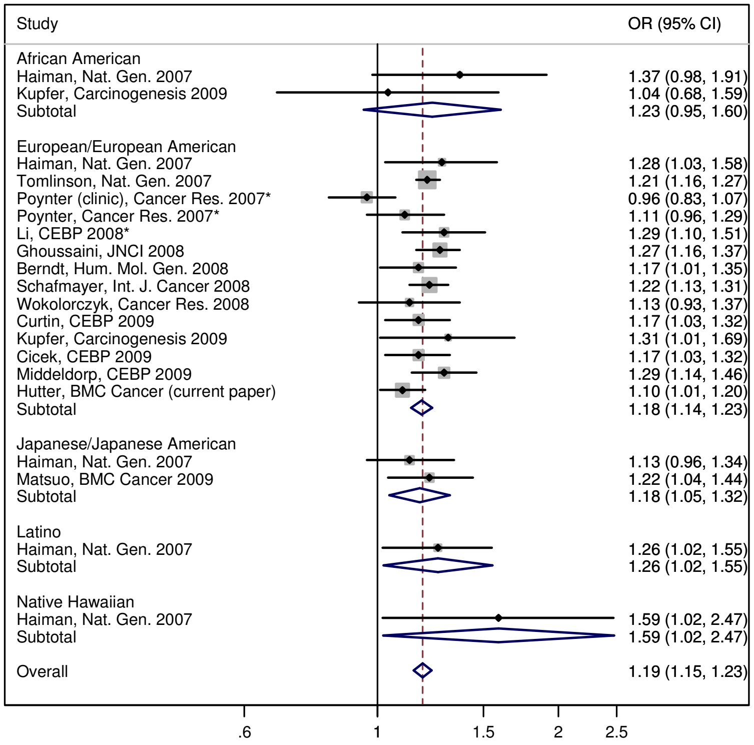

B.

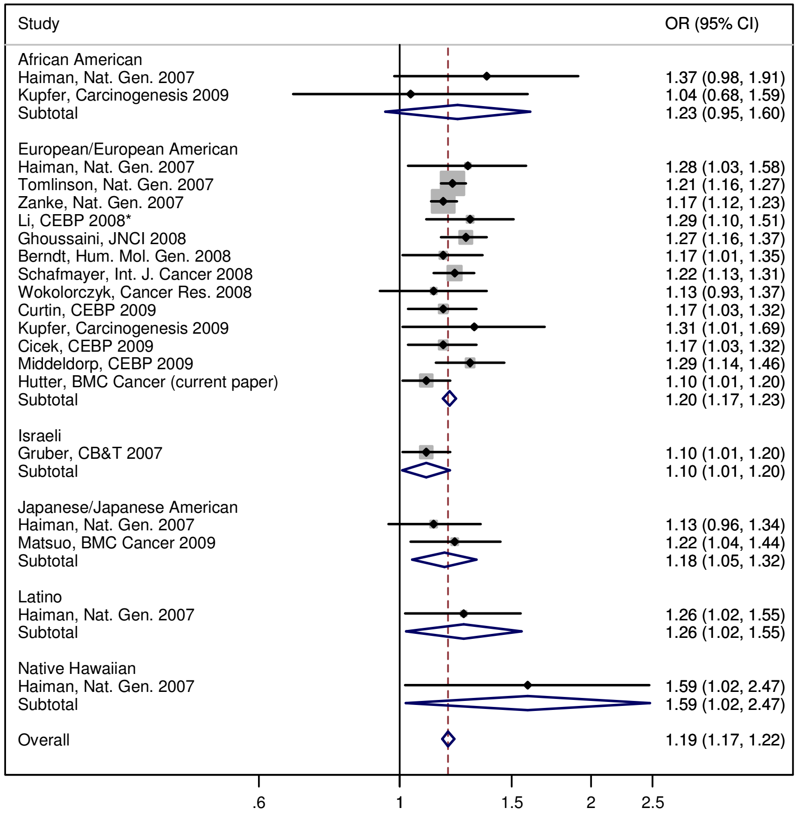

Supplemental Figure 1.

A. Forest plot of effect sizes from random-effects meta-analysis estimates of the association between colorectal cancer risk and 8q24 SNP rs6983267 including sib-pair analysis by Poynter et al. The symbol size indicates the weight for each study, and lines indicate the confidence intervals. Studies are grouped by race/ethnicity. Two studies (indicated by an asterisk) reported more than one racial/ethnicity group, but are grouped with the European/European American studies, since that is the primary group for both studies (Poynter et al reports the percentage Non-Hispanic white as 85-86% for both cases and controls and 96-97% for clinic based controls; Li reports the percentage Caucasian as 94-95% for both cases and controls). Both studies report that similar results were found when they restricted to white/Caucasian subjects.

B. Forest plot of effect sizes from random-effects meta-analysis estimates of the association between colorectal cancer risk and 8q24 SNPs rs6983267 and rs10505477. The symbol size indicates the weight for each study, and lines indicate the confidence intervals. Studies are grouped by race/ethnicity. One studies (indicated by an asterisk) reported more than one racial/ethnicity group, but is grouped with the European/European American studies, since that is the primary group reported (the percentage Caucasian as 94-95% for both cases and controls). Both studies report that similar results were found when restricted to white/Caucasian subjects.
