# Supplementary material for: Characterization of the association between 8q24 and colon cancer: gene-environment exploration and meta-analysis
Source: BMC Cancer. 2010 Dec 4;10:670. doi: 10.1186/1471-2407-10-670 (PMC3017062; doi:10.1186/1471-2407-10-670)
Supplement: Additional file 7 — Supplemental figure S2. Funnel plot and cumulative meta-analysis plots for meta-analysis. Funnel plot of odds ratio (OR) vs. standard error of OR for studies included in the meta-analysis and cumulative meta-analysis plots for rs6983267 in European/European American populations. These diagnostic plots can be used to assess biases. [file 1471-2407-10-670-S7.PDF]

A.

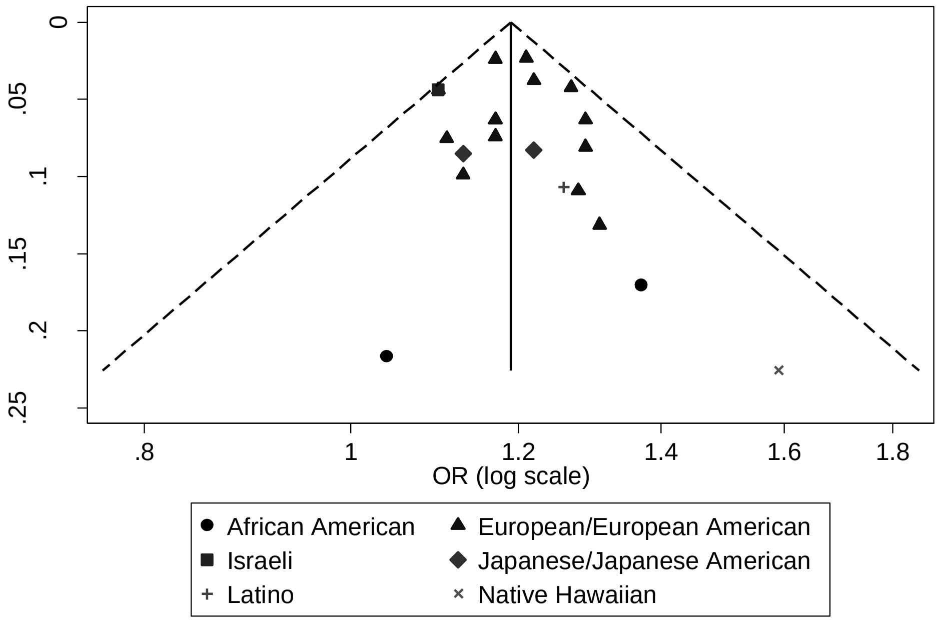

B.

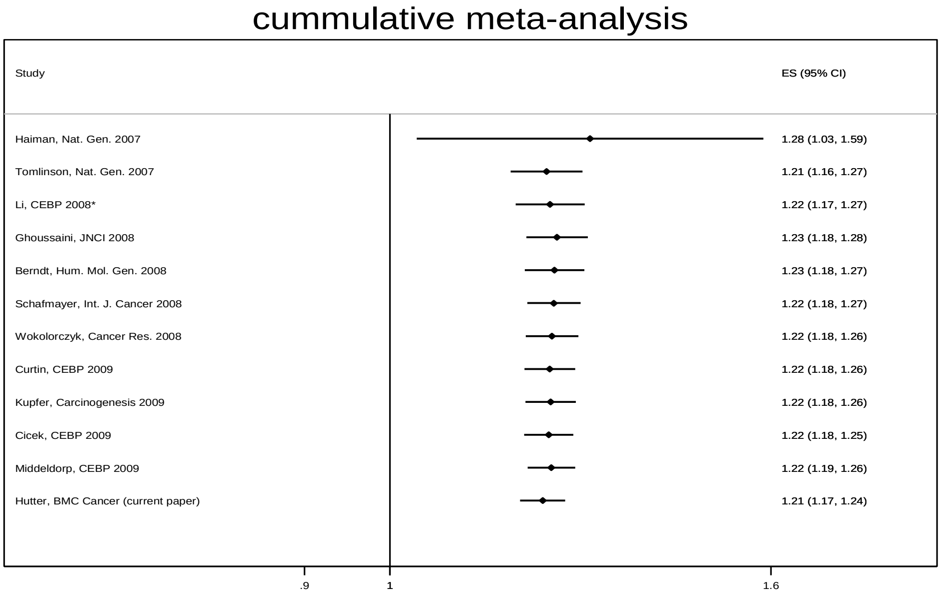

Supplemental Figure 2.

A.Funnel plot for meta-analysis of the association between colorectal cancer risk and 8q24 SNPs rs6983267 and rs10505477. Visual inspection of this plot does not indicate evidence for substantial bias. Both small and large studies fall within the expected 95% confidence interval lines and there is not evidence for asymmetry around the overall estimate.

B.Cumulative meta-analysis for association between colorectal cancer risk and 8q24 SNP rs6983267 in European/European American populations. After an initial study with a slightly higher odds ratio, the estimate stays consistent around 1.21-1.23.
